# The relationship between attributional style and destructive responses to job dissatisfaction: an exploratory study of internal migrant workers in China

**DOI:** 10.1080/21642850.2014.919864

**Published:** 2014-05-30

**Authors:** Siu-On Kwan, Fu-Keung Daniel Wong

**Affiliations:** ^a^ Department of Management, City University of Hong Kong, Kowloon Tong, Hong Kong Special Administrative Region, People's Republic of China; ^b^ Department of Applied Social Studies, City University of Hong Kong, Kowloon Tong, Hong Kong Special Administrative Region, People's Republic of China

**Keywords:** attributional style, job dissatisfaction, internal migrant workers, China, self-construal

## Abstract

This study examines the relationship between attributional style and destructive responses to job dissatisfaction among internal migrant workers in mainland China. Contrary to previous studies conducted in the West, we found that internality of bad events was negatively related to destructive responses to job dissatisfaction. Stability and globality were positively related to destructive responses to job dissatisfaction. We suggest that the concept of interdependent self-construal may explain the unique positive meaning of internality of bad events among Chinese migrant workers. The practical significance of the findings is discussed.

## Introduction

Abramson, Seligman, and Teasdale (1978), in their reformulated model of learned helplessness, propose that people have a tendency to ask why they are helpless when they face helpless situations in their lives. When people expect or learn that life outcomes are uncontrollable, they are more likely to exhibit helplessness deficits. These deficits could be expressed in the form of retarded initiation of voluntary responses and depressed affect. Abramson et al. (1978) believe that the attributional style that individuals adopt has a great impact on the generalizability and the duration of the helplessness deficits as well as their self-esteem. This study focuses on the internality dimension of attributional style which is believed to be closely related to a person's self-esteem. Based on Markus and Kitayama's (1991) conceptualization of self-construal, we propose that making an internal attribution of a bad event in life may be more prevalent among people from a culture in which interdependent self-construal is predominant. In the following, we will describe the three dimensions of attributional style, review previous work on the relationship between attributional style and performance, and propose how the internality dimension of attributional style may have a negative relationship with destructive responses to job dissatisfaction among internal migrant workers in mainland China.

Attributional style is defined as the way in which people habitually explain the cause of a bad event that has happened in their lives (Peterson & Seligman, 1984). Internality, stability, and globality are the three dimensions of attributional style. Abramson et al. (1978) claimed that internality of bad events would negatively affect self-esteem. In other words, damage can be done to a person's self-esteem when that person consistently believes that the cause of a bad event is due to his/her own actions. Stability refers to the extent to which a person habitually believes the cause of a bad event is stable across time. Globality refers to the extent to which a person habitually believes that the cause of a bad event in one area will affect other areas of their lives. In summary, people who habitually explain bad events in their lives by high internality (i.e. “the bad event was caused by me”), high stability (i.e. “the cause of the bad event will never go away”), and high globality (i.e. “the cause of the bad event will affect other areas of my life”) are said to have a pessimistic attributional style. In contrast, people with an optimistic attributional style have low levels of internality, stability, and globality in explaining bad events in their lives. They habitually think that the cause of a bad event has little to do with them, that the cause is transient, and that it will only affect one aspect of their lives.

Previous studies have found that people with a pessimistic attributional style are more likely to live under poor health conditions (Peterson, Seligman, & Valliant, 1988) and exhibit a low level of performance in schools (Peterson & Barret, 1987) and in sports (Seligman, Nolen-Hoeksema, Thornton, & Thornton, 1990). A number of studies have examined the relationship between attributional style and performance in the workplace. Employees with an optimistic attributional style were found to make more sales and were more likely to stay in an organization than those with a pessimistic attributional style (Corr & Gray, 1996; Seligman & Schulman, 1986). Employees with a pessimistic attributional style experienced more stress during job relocation and a lower level of job satisfaction (Martin, Leach, Norman, & Silvester, 2000; Welbourne, Eggerth, Hartley, Andrew, & Sanchez, 2007).

More recently, Hui, Pak, Kwan, and Chao (2012) studied the relationship between attributional style and responses to job dissatisfaction among Chinese employees in Hong Kong. They measured both constructive responses and destructive responses to job dissatisfaction. Constructive responses refer to voice and loyalty, while destructive responses refer to exit and neglect (Farrell, 1983). In the first part of their study, Hui et al. (2012) could not find any relationship between internality and destructive responses to job dissatisfaction. In the second part of their study, however, Hui et al. (2012) found that internality was negatively related to destructive responses to job dissatisfaction. Taken together, their findings contradict previous research conducted in the West, which typically found a negative relationship between internality of bad events and positive performance indicators in the workplace. Due to the very limited number of studies on the relationship between attributional style and job performance within the Chinese population, whether internality is negatively related to destructive responses to job dissatisfaction among Chinese employees remains largely unclear. By examining this relationship in this study with a sample of internal migrant workers in mainland China, we hope to shed more light on this important question.

## Internality as perceived by Chinese workers

In this study, we propose that internality of negative events could have a favorable impact on job performance based on the theoretical framework of self-construals by Markus and Kitayama (1991). These researchers theorized two divergent construals of the self among different cultures, namely independent self-construal and interdependent self-construal. Markus and Kitayama (1991) suggest that self-construal may affect a person's intrapersonal and interpersonal processes. Independent self-construal involves perceiving the self as an autonomous, whole, and independent entity, which does not require other people, in the formation of self-esteem. In this regard, responses to the social environment are motivated by a want to express or assert internal self-attributes, such as abilities, opinions, judgments, and personality characteristics. Markus and Kitayama (1991) assumed people from Western cultures would predominantly have an independent self-construal.

In contrast, people with an interdependent self-construal focus on the fundamental relatedness of individuals in a social context. In this instance, the self becomes meaningful when it is perceived within a social relationship; the self is only complete with the inclusion of others. The self-esteem of people with interdependent self-construal largely depends on their ability to attend to others, to adjust themselves to and maintain harmony with others in a social context. Thus, they are motivated to fit in and achieve a sense of belonging to a social group. Markus and Kitayama (1991) contend that people in the Chinese culture are more likely than people in Western cultures to adopt an interdependent self-construal. Thus, having a high internality of bad events (i.e. blaming one's self for bad events) may enhance Chinese employees self-esteem because doing so can help in attending to other people's needs and maintaining harmonious relationships in the workplace in the Chinese culture. This improved self-esteem could in turn lead to better performance at work.

## Internal migrant workers in China

In this study, we targeted internal migrant workers in China because these employees are very likely to experience situations of helplessness in their lives and also due to the fact that they constitute a considerable proportion of the total workforce in China. Chinese migrant workers in this study are defined as internal migrants who have been given the legal right to work temporarily in certain cities in China. These internal migrants moved from rural areas to different Chinese cities. Many of them came from the western regions of China and congregated in eastern and southern coastal cities, such as Beijing, Shanghai, Guangdong, and Fujian. According to a recent report by the National Bureau of Statistics of China (2012), there were 262 million rural-to-urban migrants in China, accounting for about one-fifth of the country's total population.

Many of the migrant workers are in search of a better living standard for themselves and their families left behind at home. However, these migrant workers are very likely to face situations of helplessness when they begin working in cities. In China, due to the *Hukou* system (the household registration system), workers who were not born in a particular city cannot register as official residents and therefore are not entitled to subsidized housing, education, social security, or medical benefits. Rather, they tend to live in poorly sanitized and usually overcrowded dormitories provided by their employers or in shared accommodations. Most migrant workers take up physically demanding jobs which local residents disdain, such as manual labor and factory and service work (Wong, Chang, & He, 2007). Since the majority of these internal migrant workers are uneducated and do not have special skills, job mobility among them is very low. Coupled with a lack of knowledge of their legal rights, these internal migrant workers have been subjected to a great deal of exploitation, such as unreasonably long working hours and lack of employee benefit schemes. Some internal migrant workers are put in hazardous environments with dust, toxic substances, noise, and poor ventilation (Wong et al., 2007). The recent tragic incident of 13 suicides in one of China's largest factories, Foxconn, has brought to light the difficulties faced by migrant workers in China's cities. Indeed, the harsh realities and unfulfilled expectations experienced in the cities might have created a group of internal migrant workers who have the potential risk of developing a sense of helplessness. Thus, internal migrant workers in China are an appropriate population in which to study the relationship between attributional style and work performance in China.

To reiterate, we proposed that internality of bad events would be prevalent among Chinese migrant workers who predominantly have an interdependent self-construal. Thus, we expect that the internality of bad events would be negatively related to destructive responses in job dissatisfaction. Therefore,

Hypothesis 1: The internality of bad events will be negatively related to destructive responses to job dissatisfaction among Chinese workers.

Previous studies on attributional style have shed some light on the possible relationship between stability/globality of bad events and performance indicators in the workplace. Furnham, Sadka, and Brewin (1992) found a negative relationship between stability/globality and job motivation among British employees. Hui et al. (2012) found a positive relationship between the composite scores of stability and globality and destructive responses to job dissatisfaction among Chinese employees in Hong Kong. Other studies, however (Corr & Gray, 1996; Martin et al., 2000; Seligman & Schulman, 1986), reported only a composite score of attributional style by averaging internality, stability, and globality. Thus, it is difficult to identify the possible relationship between stability/globality and job performance. In a non-work context, Sweeney, Anderson, and Bailey (1986) conducted a meta-analysis of over 100 studies involving 150,000 participants (predominantly people from the West) on the relationship between attributional style and depression. They found that stability and globality were related to depression with an effect size of 0.20 and 0.22, respectively. Based on the review of the literature above, we believe that employees who show high stability *and* high globality of bad events are less likely to perform well in the workplace when compared to employees displaying low stability and low globality. Since we could not identify any psychological factors or cross-cultural factors which would greatly affect the perception of stability and globality by Chinese workers as compared with people in the West, we expect the relationship of a composite score of stability and globality with performance among the Chinese workers to be very similar to that previously found in the literature. Therefore,

Hypothesis 2: The composite of stability and globality of bad events will be positively related to destructive responses to job dissatisfaction among Chinese workers.

## Method

### Participants

“Migrant workers” refers to internal, rural migrants who have been given the legal right to work temporarily in certain cities in China. The inclusion criteria for this study were: (1) participants had to be living and working in cities temporarily without a local residence status and (2) participants to be aged 18 and above. Shanghai was chosen as the study site as it has been found to be the largest host city for internal migrant workers in mainland China (State Council, 2004). This study used a convenience sampling method to choose its subjects. According to the Shanghai municipal regulations on immigrants, all legal internal migrant workers must register with the local residents' committee where they are living to receive and renew temporary residency certificates. Thus, all local residents' committees have a name-list of internal migrants. Two residents' committees from areas where many internal migrant workers usually live were chosen. Three hundred and thirty-two internal migrant workers initially agreed to participate in the research. Two research assistants, undergraduate students at the School of Social Work of a local university in Shanghai, were employed and trained to call each migrant worker to seek their formal consent; 261 internal migrants confirmed their participation. However, during actual face-to-face data collection, 59 potential participants declined and thus a total of 202 internal migrant workers finally completed the questionnaire. Such a relatively high refusal rate was unfortunate; the possibility exists that some internal migrant workers were very sensitive toward the intentions of the interviewers and therefore did not cooperate in the research. In the end, most questionnaires were completed at the migrant workers' homes and most completed their questionnaires independently. A few respondents completed the questionnaire with the help of the research assistants. The participants' demographic information are shown in Table 1. The national profile of Chinese migrant workers is given in another column in the same table. It can be seen that the composition of this sample is similar to the national profile in terms of gender, age, and educational level.

**Table 1.** Demographics of participants in comparison with the national profile.

		*N*	Sample %	National %
Gender	Male	102	50.5	66.4
	Female	81	40.1	33.6
	Missing	19	9.4	0
Age	20 or below	8	4	4.9
	21–30	60	29.7	31.9
	31–40	53	26.2	22.5
	41–50	60	29.7	25.6
	51–60	9	4.5	15.1
	Missing	12	5.9	0
Household registration type	Urban	35	17.3	N/A
	Rural	155	76.7	N/A
	Missing	12	5.9	N/A
Educational level	Illiterate	0	0	1.5
	Junior high or below	132	65.3	74.8
	Senior high school or above	57	28.2	23.7
	Missing	13	6.4	N/A
Marital status	Single	27	13.4	N/A
	Married	164	81.2	N/A
	Missing	11	5.4	N/A
Total		202	100	

Note: Data on national statistics were obtained from the National Bureau of Statistics of China (2012).

## Measures

### Attributional style

Attributional style reflects individual difference in terms of the causal attribution a person habitually makes to explain the cause of a bad event. The three dimensions of attributional style are internality, stability, and globality. A validated measure of the attributional style of Chinese people was adopted (based on Hui et al., 2012). To assess how a person would explain the cause of five hypothetical bad events, the dimensions of attributional style were measured with a seven-point Likert scale. The five bad events were: (1) having a serious argument with a family member; (2) being cheated during a purchase; (3) failing a school test or some job assignment; (4) having a conflict with a friend or colleague; and (5) not being able to complete a job assignment on time. These events were adapted from previous validated instruments of attributional style (Dykema, Bergbower, Doctora, & Peterson, 1996; Peterson et al., 1988). Composite 2 was obtained by taking the average score of stability and globality across the five events, while Composite 3 was calculated by taking the average score of internality, stability, and globality across the same events. Internal consistency for internality, stability, globality, Composite 2, and Composite 3 were 0.64, 0.71, 0.78, 0.84, and 0.79 respectively, which were found to be reasonable and similar to those found in previous work.

### Destructive responses to job dissatisfaction

“Destructive responses to job dissatisfaction” refers to an employee's intention to quit his/her job and/or neglectful attitude in the workplace (Farrell, 1983). Destructive responses to job dissatisfaction were measured with six items which were adopted from Rusbult, Farrell, Roger, and Mainous (1988). The items measured were exit intentions (e.g. “I want to quit the job as soon as possible”) and neglectful behavior (e.g. “I now care little about my job performance”). Internal consistency was at an acceptable level (Cronbach's alpha = 0.63).

## Data analyses

The data were first cleaned by running some descriptive statistics to ensure that they were not out of range. In case of doubt, the original questionnaires were checked for accuracy during data entry. IBM SPSS Statistics (version 21) was used as a tool for data analyses, such as descriptive statistics, correlations, and regressions. Three hierarchical regression analyses were undertaken to explore the magnitude and the direction of the predictive power of internality on destructive responses to job dissatisfaction.

## Results

As shown in the correlation analyses in Table 2, internality of bad events did not correlate with destructive responses to job dissatisfaction. In addition, internality did not correlate with stability or globality at all. It seems that internality of bad events carried a unique meaning for the Chinese workers. On the other hand, stability, globality, and Composite 2 correlated positively with destructive responses to job dissatisfaction. This finding is consistent with the results of the studies by Hui et al. (2012).

**Table 2.** Means, standard deviations, reliabilities, and correlations of variables (*n* = 190 after list-wise deletion).

			Correlation with attributional style				
	Mean	SD	Internality	Stability	Globality	Composite 2	Composite 3
Internality	3.95	1.23	–	−0.04^ns^	−0.03^ns^	−0.04^ns^	0.42***
Stability	3.16	1.33	–	–	0.68***	0.91***	0.81***
Globality	3.25	1.40	–	–	–	0.92***	0.83***
Composite 2	3.21	1.25	–	–	–	–	0.89***
Composite 3	3.46	0.92	–	–	–	–	–
Gender^a^	0.44	0.50	0.02^ns^	–0.08^ns^	−0.14^ns^	−0.12^ns^	−0.10^ns^
Age	35.69	9.70	−0.03^ns^	0.09^ns^	0.08^ns^	0.10^ns^	0.07^ns^
Household registration type^b^	0.82	0.39	0.04^ns^	0.15*	0.01^ns^	0.09^ns^	0.10^ns^
Educational level^c^	0.30	0.46	−0.02^ns^	–0.06^ns^	–0.07^ns^	−.07^ns^	−0.07^ns^
Marital status^d^	0.86	0.35	−0.07^ns^	–0.08^ns^	–0.09^ns^	−0.09^ns^	−0.11^ns^
*Dependent variable*							
Destructive responses to job dissatisfaction	2.47	0.72	−0.07^ns^	0.20**	0.23**	0.23**	0.18*

Notes: Composite 2 is an average of stability and globality. Composite 3 is an average of internality, stability, and globality. ns, non-significant.

^a^Gender: 0 = male and 1 = female.

^b^Household registration type: 0 = urban registration and 1 = rural registration.

^c^Educational level: 0 = junior high or below and 1 = senior high or above.

^d^Marital status: 0 = single and 1 = married.

*p < .05, two-tailed.

**p < .01, two-tailed.

***p < .001, two-tailed.

The predictive power of internality toward destructive responses to job dissatisfaction was explored with the use of hierarchical regression analyses (Table 3). Demographic variables, such as gender, age, household registration type, education, and marital status were entered in Step 1 of the regression analyses to control for a possible effect on destructive responses to job dissatisfaction. It was found that these demographic variables had no significant relationship with destructive responses to job dissatisfaction. Following practices undertaken in previous research, we entered Composite 3, which is the average of internality, stability, and globality, into the regression equation. After controlling for the possible effects of the demographic variables, Composite 3 was found to account for 3% of the variance (Δ*R* ^2^, *p* < .05, two-tailed) in destructive responses to job dissatisfaction. Consistent with previous research findings on attributional style (Corr & Gray, 1996; Martin et al., 2000; Seligman & Schulman, 1986; Welbourne et al., 2007), Composite 3 was found to be positively related to performance indicators, such as destructive responses to job dissatisfaction (*β* = 0.19, *p* < .05, two-tailed).

**Table 3.** Summary of regression results predicting destructive responses to job dissatisfaction (*n* = 136 after list-wise deletion).

		Destructive responses to job dissatisfaction		
		*B*	SE *B*	*β*
*By Composite 3*				
Step 1	Gender	−0.12	0.13	−0.09^ns^
	Age	0.01	0.01	0.14^ns^
	Registration type	0.14	0.17	0.07^ns^
	Education	0.07	0.13	0.05^ns^
	Marital status	−0.08	0.20	−0.04^ns^
Step 2	Composite 3	0.14	0.07	0.19*
	*R*^2^ for Step 1	0.03		
	△*R*^2^ for Step 2	0.03*		
	Final *R*^2^ (*η*^2^)	0.06		
	Adjusted *R*^2^ (*η*^2^)	0.02		
*By Composite 2 and internality*				
Step 1	Gender	−0.12	0.13	−0.09^ns^
	Age	0.01	0.01	0.14^ns^
	Registration type	0.14	0.17	0.07^ns^
	Education	0.07	0.13	0.05^ns^
	Marital status	−0.08	0.20	−0.04^ns^
Step 2	Composite 2	0.18	0.05	0.31***
	Internality	−0.10	0.05	−0.18*
	*R*^2^ for Step 1	0.03		
	△*R*^2^ for Step 2	0.11***		
	Final *R*^2^ (*η*^2^)	0.14		
	Adjusted *R*^2^ (*η*^2^)	0.09		
*By internality, stability, and globality*				
Step 1	Gender	−0.12	0.13	−0.09^ns^
	Age	0.01	0.01	0.14^ns^
	Registration type	0.14	0.17	0.07^ns^
	Education	0.07	0.13	0.05^ns^
	Marital status	−0.08	0.20	−0.04^ns^
Step 2	Internality	−0.10	0.05	−0.18*
	Stability	0.08	0.06	0.14
	Globality	0.11	0.06	0.20
	*R*^2^ for Step 1	0.03		
	△*R*^2^ for Step 2	0.11**		
	Final *R*^2^ (*η*^2^)	0.14		
	Adjusted *R*^2^ (*η*^2^)	0.08		

Notes: Coefficients shown here are from the final model. Intercepts are omitted. Composite 2 is an average of stability and globality. Composite 3 is an average of internality, stability, and globality. ns, non-significant.

*p < .05, two-tailed.

**p < .01, two-tailed.

***p < .001, two-tailed.

Since we theorized that internality may have a different meaning to Chinese migrant workers who are likely to develop helplessness, we entered Composite 2 (an average of stability and globality) and internality into another regression analysis after controlling for the demographic variables. We found that Composite 2 and internality together account for 11% of the variance (Δ*R* ^2^, *p* < .001, two-tailed) in destructive responses to job dissatisfaction. The amount of variance explained by Composite 2 and internality is much greater than that explained by Composite 3 (11% vs. 3%). In addition, Composite 2 is positively related to destructive responses to job dissatisfaction (*β* = 0.31, *p* < .001, two-tailed). To investigate the possible effects of stability and globality on destructive responses to job dissatisfaction separately, we entered internality, stability, and globality into the last regression analysis. However, we did not find any relationship between stability or globality and destructive responses to job dissatisfaction. Contrary to findings in the West, we found that internality was negatively related to destructive responses to job dissatisfaction (*β* = −.18; *p* < .05, two-tailed). In short, the regression analyses provide good support for both Hypothesis 1 and Hypothesis 2.

## Discussion

This study aimed at examining the relationship between attributional style and destructive responses to job dissatisfaction among internal migrant workers in mainland China. An interesting finding of this study is that internality of a bad event is negatively related to destructive responses to job dissatisfaction. In other words, Chinese migrant workers who take the blame for bad events in their lives are less likely to quit their job or to show neglectful behavior in the workplace. This study, to the best of our knowledge, is the first to investigate the relationship between attributional style and performance indicators among Chinese workers in mainland China. Specifically, we chose Chinese internal migrant workers as participants because they are more likely to develop learned helplessness at work due to poor working conditions and lack of social support.

By integrating a reformulated model of learned helplessness (Abramson et al., 1978) and the concept of self-construal (Markus & Kitayama, 1991), we put forward the theoretical proposition that making an internal attribution of a bad event may be compatible with Chinese employees' largely interdependent self-construal. Thus, internality of a bad event may be associated with better performance among Chinese employees. In addition, Confucianism may be another cultural construct that contributes to the positive perception of internality of bad events among Chinese workers. Under the influence of Confucianism, Chinese people espouse the values of modesty, shame, and hierarchy of relationship (Chinese Cultural Connection, 1987). Thus, Chinese people may be more likely to respect authority and to be more receptive than their counterparts in Western cultures to accept criticism from others. When bad events happen in their lives, Chinese people may feel more comfortable accepting negative comments from others and bearing the blame for negative outcomes.

This study has practical implications for the selection and training of internal migrant workers in China. A validated questionnaire on attributional style could be administered to potential employees (i.e. inland Chinese migrant workers) who are looking for a job in major Chinese cities. In predicting an applicants' performance in the workplace, we recommend the use of internality and Composite 2 (i.e. the average of stability and globality) since this study found that internality and Composite 2 have a higher predictive power than the typical Composite 3 score (i.e. the average of internality, globality, and stability). With regard to training, we suggest that Chinese migrant workers with high stability and high globality of bad events undergo cognitive-behavioral training similar to that conducted by Proudfoot, Corr, Guest, and Gray (2001). Training could cover topics such as goal setting, time management, thinking errors, and project management. It is expected that this kind of training will help the workers develop a less stable and less global attribution of bad events which in turn may enhance job performance in the future.

Like other studies, this study has limitations. First, as all data were collected at one time point with the use of the same questionnaire, common method variance between the predictor and the criterion variables may have occurred (Podsakoff, MacKenzie, Lee, & Podsakoff, 2003). Second, our sample may not be representative of all Chinese migrant workers since all the respondents were recruited in Shanghai. To address these limitations, future research should collect attributional style and performance data at different time points. In addition, migrant workers from different cities could be recruited in future studies. We hope this study will stimulate further research on the relationship between attributional style and job performance in non-Western cultures.

## References

[CIT0001] Abramson L. Y., Seligman M. E. P., Teasdale J. (1978). Learned helplessness in humans: Critique and reformulation. *Journal of Abnormal Psychology*.

[CIT0002] Chinese Cultural Connection. (1987). Chinese values and the search for culture-free dimensions of culture. *Journal of Cross-Cultural Psychology*.

[CIT0003] Corr P. J., Gray J. A. (1996). Attributional style as a personality factor in insurance sales performance in the UK. *Journal of Occupational and Organizational Psychology*.

[CIT0004] Dykema J., Bergbower K., Doctora J. D., Peterson C. (1996). An attributional style questionnaire for general use. *Journal of Psychoeducational Assessment*.

[CIT0005] Farrell D. (1983). Exit, voice, loyalty, and neglect as responses to job dissatisfaction: A multidimensional scaling study. *Academy of Management Journal*.

[CIT0006] Furnham A., Sadka V., Brewin C. R. (1992). The development of an occupational attributional style questionnaire. *Journal of Organizational Behavior*.

[CIT0007] Hui C. H., Pak S. T., Kwan S., Chao A. (2012). Attributional style and engagement/disengagement responses in the Chinese workforce. *Applied Psychology: An International Review*.

[CIT0008] Markus H. R., Kitayama S. (1991). Culture and the self: Implications for cognition, emotion, and motivation. *Psychological Review*.

[CIT0009] Martin R., Leach D. J., Norman P., Silvester J. (2000). The role of attributions in psychological reactions to job relocation. *Work & Stress*.

[CIT0010] National Bureau of Statistics of China. (2012). http://www.stats.gov.cn/tjfx/jdfx/t20130527_402899251.htm.

[CIT0011] Peterson C., Barret L. C. (1987). Explanatory style and academic performance among university freshmen. *Journal of Personality and Social Psychology*.

[CIT0012] Peterson C., Seligman M. E. P. (1984). Causal explanations as a risk factor for depression: Theory and evidence. *Psychological Review*.

[CIT0013] Peterson C., Seligman M. E. P., Valliant G. (1988). Pessimistic explanatory style is a risk factor for physical illnesses: A thirty-five-year longitudinal study. *Journal of Personality and Social Psychology*.

[CIT0014] Podsakoff P. M., MacKenzie S. B., Lee J., Podsakoff N. P. (2003). Common method biases in behavioral research: A critical review of the literature and recommended remedies. *Journal of Applied Psychology*.

[CIT0015] Proudfoot J. G., Corr P. J., Guest D. E., Gray J. A. (2001). The development and evaluation of a scale to measure occupational attributional style in the financial service sector. *Personality and Individual Differences*.

[CIT0016] Rusbult C. E., Farrell D., Rogers G., Mainous A. G. (1988). Impact of exchange variables on exit, voice, loyalty, and neglect: An integrative model of responses to declining job satisfaction. *Academy of Management Journal*.

[CIT0017] Seligman M. E. P., Nolen-Hoeksema S., Thornton N., Thornton K. M. (1990). Explanatory style as a mechanism of disappointing athletic performance. *Psychological Science*.

[CIT0018] Seligman M. E. P., Schulman P. (1986). Explanatory style as a predictor of productivity and quitting among life insurance sales agents. *Journal of Personality and Social Psychology*.

[CIT0019] State Council. (2004).

[CIT0020] Sweeney P. D., Anderson K., Bailey S. (1986). Attributional style in depression: A meta-analytic review. *Journal of Personality and Social Psychology*.

[CIT0021] Welbourne J. L., Eggerth D., Hartley T. A., Andrew M. E., Sanchez F. (2007). Coping strategies in the workplace: Relationships with attributional style and job satisfaction. *Journal of Vocational Behavior*.

[CIT0022] Wong F. D., Chang Y. L., He X. S. (2007). Rural migrant workers in urban China: Living a marginalized life. *International Journal of Social Welfare*.

